# The Utility of C-Reactive Protein, Procalcitonin, and Leukocyte Values in Predicting the Prognosis of Patients with Pneumosepsis and Septic Shock

**DOI:** 10.3390/medicina60101560

**Published:** 2024-09-24

**Authors:** Melek Doganci, Guler Eraslan Doganay, Hilal Sazak, Ali Alagöz, Mustafa Ozgur Cirik, Derya Hoşgün, Emine Banu Cakiroglu, Murat Yildiz, Maside Ari, Tarkan Ozdemir, Derya Kizilgoz

**Affiliations:** Department of Anesthesiology and Reanimation, Ankara Ataturk Sanatorium Training and Research Hospital, University of Health Sciences, 06290 Ankara, Turkey; gulerdoganay@hotmail.com.tr (G.E.D.); hilalgun@yahoo.com (H.S.); mdalagoz@gmail.com (A.A.); dr.ozgurr@hotmail.com (M.O.C.); deryahosgun@gmail.com (D.H.); banu.cakiroglu@yahoo.com (E.B.C.); drmuratyildiz85@gmail.com (M.Y.); masidetuten@icloud.com (M.A.); tarkanozdemir78@gmail.com (T.O.); deryaozaydin01@hotmail.com (D.K.)

**Keywords:** C-reactive protein, leukocyte, pneumosepsis, procalcitonin, septic shock

## Abstract

*Background and Objectives:* The predictive value of changes in C-reactive protein (CRP), procalcitonin, and leukocyte levels, which are commonly used in the diagnosis of infection in sepsis and septic shock, remains a topic of debate. The aim of this study was to evaluate the effectiveness of changes in CRP, procalcitonin, and leukocyte counts on the prognosis of 230 patients admitted to the intensive care unit (ICU) with the diagnosis of sepsis and pneumonia-related septic shock between 1 April 2022 and 31 December 2023, and to investigate whether any of these markers have a superior predictive value over the others in forecasting prognosis. *Materials and Methods:* This single-center, retrospective, cross-sectional observational study included patients who developed sepsis and septic shock due to community-acquired pneumonia and were admitted to the ICU. Demographic data, 1-month and 90-day mortality rates, length of stay in the ICU, discharge to the ward or an outside facility, need for dialysis after sepsis, need for invasive or noninvasive mechanical ventilation during the ICU stay and the duration of this support, whether patients admitted with sepsis or septic shock required inotropic agent support during their stay in the ICU and whether they received monotherapy or combination therapy with antibiotics during their admission to the ICU, the Comorbidity Index score (CCIS), CURB-65 score (confusion, uremia, respiratory rate, BP, age ≥ 65), and Acute Physiology and Chronic Health Evaluation II (APACHE-II) score were analyzed. Additionally, CRP, procalcitonin, and leukocyte levels were recorded, and univariate and multivariate logistic regression analyses were performed to evaluate their effects on 1- and 3-month mortality outcomes. In all statistical analyses, a *p*-value of <0.05 was accepted as a significant level. *Results:* According to multivariate logistic regression analysis, low BMI, male gender, and high CCIS, CURB-65, and APACHE-II scores were found to be significantly associated with both 1-month and 3-month mortality (*p* < 0.05). Although there was no significant relationship between the first-day levels of leukocytes, CRP, and PCT and mortality, their levels on the third day were observed to be at their highest in both the 1-month and 3-month mortality cases (*p* < 0.05). Additionally, a concurrent increase in any two or all three of CRP, PCT, and leukocyte values was found to be higher in patients with 3-month mortality compared with those who survived (*p* = 0.004). *Conclusions:* In patients with pneumoseptic or pneumonia-related septic shock, the persistent elevation and concurrent increase in PCT, CRP, and leukocyte values, along with male gender, advanced age, low BMI, and high CCIS, CURB-65, and APACHE-II scores, were found to be significantly associated with 3-month mortality.

## 1. Introduction

Sepsis is a life-threatening organ dysfunction caused by a dysregulated host response to infection [1]. Septic shock is a critical condition characterized by persistent hypotension and tissue hypoperfusion due to underlying circulatory, cellular, and metabolic abnormalities, despite adequate fluid resuscitation, with symptoms varying depending on the affected system [2].

The Sequential Organ Failure Assessment (SOFA) score is used to calculate the degree of organ dysfunction in sepsis. According to the most recently updated sepsis guidelines, although the calculation of the SOFA score as a screening tool seems reasonable, prospective validation of its usefulness at the bedside is lacking. Sepsis screenings such as the Quick Sepsis-Related Organ Failure Assessment (qSOFA), systemic inflammatory response syndrome criteria (SIRS), Modified Early Warning Score (MEWS), and National Early Warning Score (NEWS) are also limited [1].

Pneumonia is one of the leading causes of sepsis. Studies have shown that sepsis related to pneumonia is associated with higher mortality rates compared with sepsis caused by other infections [3].

Procalcitonin (PCT) is a calcitonin precursor hormone that is undetectable in healthy individuals. However, it provides additional information that can support clinical and diagnostic parameters and influence antibiotic duration. PCT is used as a biomarker in various inflammatory conditions such as sepsis, severe burns, acute pancreatitis, and postoperative infections. C-reactive protein (CRP), produced in response to specific pro-inflammatory cytokines, is another crucial marker of inflammation. It is widely used to assess the prognosis of patients with conditions like acute pancreatitis, pulmonary infections, malignant tumors, and gout arthritis, reflecting the severity of the disease [4].

Leukocytes have a critical effect on the immune system, and in sepsis, disruption of the endothelial barrier, usually caused by leukocyte–endothelial cell (EC) interactions, causes organ damage [5]. Recent sepsis studies utilizing transcriptomic analysis have provided evidence of significant changes in the leukocyte transcriptomes of septic patients. The leukocytes of intensive care unit (ICU) patients show a highly altered transcriptome, with 70–80% of all RNA transcripts being significantly different [6]. Particularly in critically ill patients with community-acquired pneumonia, transcriptomic analysis of circulating leukocytes has identified an immunosuppressive state associated with a higher mortality rate in the sepsis response [7]. Such studies support the understanding that sepsis can lead to profound and persistent immunosuppression.

PCT, CRP, and leukocyte count are among the most commonly used biomarkers to predict sepsis and septic shock [8]. While there are limited data on the prognostic role of these biomarkers, some studies have emphasized that changes in PCT and CRP levels can be indicative of the outcome of sepsis [9]. However, there is no clear consensus in the literature regarding the superiority of one biomarker over the others. The potential of CRP, PCT, and leukocyte count changes, either individually or in combination, to predict prognosis in patients with sepsis and septic shock remains a subject of interest. We believe that a dynamic approach to evaluating these biological markers may provide more insight into the outcomes of patients with sepsis. This study aims to assess the impact of changes in CRP, PCT, and leukocyte counts on the prognosis of patients admitted to the ICU with a diagnosis of pneumosepsis and pneumonia-related septic shock and to explore whether any of these markers have a superior predictive value in forecasting prognosis.

## 2. Materials and Methods

This single-center, retrospective, cross-sectional observational study included 230 patients admitted to the adult tertiary ICU at the University of Health Sciences Ankara Atatürk Sanatorium Training and Research Hospital with a diagnosis of pneumosepsis and pneumonia-related septic shock between 1 April 2022 and 31 December 2023. After obtaining approval from the institution’s Scientific Studies Ethics Committee, with approval number 2024-BÇEK/57 dated 24 April 2024, the hospital information management system records and files of the patients were retrospectively reviewed between 25 April 2024 and 16 June 2024.

According to the Turkish Thoracic Society’s Consensus Report on the Diagnosis and Treatment of Community-Acquired Pneumonia in Adults [10], patients diagnosed with community-acquired pneumonia who developed sepsis and septic shock related to pneumonia and were admitted to the ICU were included in this study. The inclusion criteria required patients to be over 18 years of age, to have been monitored in the ICU for more than 24 h, and to have received standard sepsis treatment according to the sepsis guidelines. The diagnosis and treatment of sepsis and septic shock followed the 2021 Surviving Sepsis Campaign guidelines [1].

**Figure 1:**
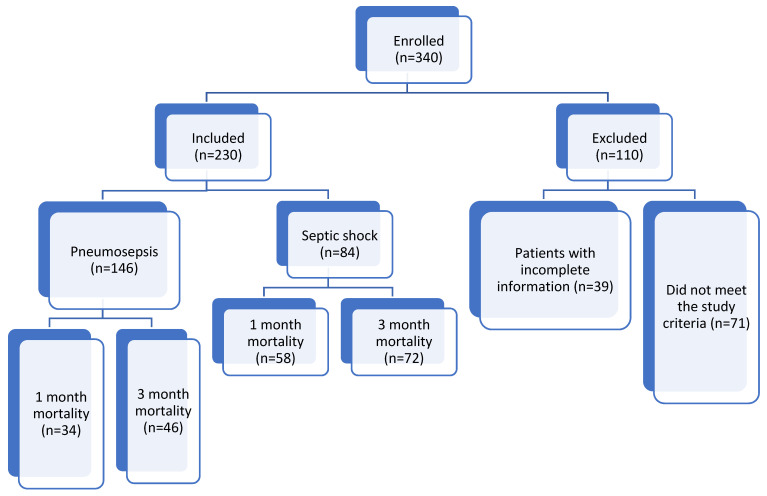
Flowchart.

The exclusion criteria for this study included patients under 18 years of age, patients who stayed in the ICU for less than 24 h, patients with extrapulmonary infections, immunocompromised patients (such as those with hematological malignancies, HIV, and neutropenia < 1000 cells/mL, or those who received chemotherapy or radiotherapy within the previous 45 days), patients with additional lung pathology, patients with rheumatologic diseases, patients with non-infectious causes of high inflammatory states such as pancreatitis, trauma, or major surgery, and patients with incomplete serum biomarker data (Figure 1).

The hospital clinical data system (AKGUN central access system) and patient ICU files were examined to access patient information. The information was collected by the attending clinician using a paper case report form. This study recorded various patient data, including age, gender, body mass index (BMI), comorbidities, whether the patients were in sepsis or septic shock at the time of ICU admission, 1-month and 90-day mortality rates, length of ICU stay, discharge status to either a ward or an external facility, need for dialysis post-sepsis, need for invasive or non-invasive mechanical ventilation (MV) during the ICU stay and the duration of such support, whether patients admitted with sepsis or septic shock required inotropic agent support during their ICU stays (continuous infusion of norepinephrine and/or dopamine was given as inotropic agents to patients with hypotension), and whether they received monotherapy or combination therapy with antibiotics at the time of ICU admission.

The severity of the illness was assessed using the CURB-65 score [confusion; blood urea nitrogen (BUN) > 20 mg/dL or urea > 42.8 mg/dL; respiratory rate ≥ 30 breaths/min; systolic blood pressure < 90 mmHg or diastolic ≤ 60 mmHg; and age ≥ 65 years], evaluated prior to ICU admission. Additionally, the Acute Physiology and Chronic Health Evaluation II (APACHE-II) score, Sequential Organ Failure Assessment (SOFA) score, and Charlson Comorbidity Index score (CCIS) were evaluated within the first 24 h after ICU admission.

The levels of CRP, PCT, and leukocytes were recorded on the day of ICU admission and subsequently on days 3, 7, and 10 of ICU follow-up. Other laboratory parameters recorded at the time of admission included hemoglobin, hematocrit, platelet count, glomerular filtration rate (GFR), creatinine, urea, alanine aminotransferase (ALT), aspartate aminotransferase (AST), total bilirubin, and albumin levels.

In order to evaluate the effects of changes in CRP, PCT, and leukocyte levels measured on the 1st, 3rd, 7th, and 10th days during ICU follow-up of patients admitted to the ICU with the diagnosis of sepsis and septic shock due to pneumonia on prognosis and to investigate the superiority of any of these markers in predicting prognosis, the measured CRP, PCT, and leukocyte levels were compared with other parameters. The relationship between CRP, PCT, and leukocyte levels measured on the 1st, 3rd, 7th, and 10th days and 1-month mortality and 3-month mortality was evaluated. To determine whether the combined elevation of CRP, PCT, and leukocyte levels or their individual elevations were more significant for mortality, the reference ranges of our hospital were used as a baseline. According to our hospital’s reference ranges, a CRP level > 5 mg/L was considered elevated CRP, a PCT level > 0.25 ng/mL was considered elevated PCT, leukocytosis was defined as a leukocyte count > 12 × 10^3^/µL, and leukopenia was defined as a leukocyte count < 4 × 10^3^/µL.

### Statistical Analysis

Data analyses were performed by using SPSS for Windows, version 22.0 (SPSS Inc., Chicago, IL, USA). Whether the distribution of continuous variables was normal or not was determined by the Kolmogorov–Smirnov test. The Levene test was used for the evaluation of homogeneity of variances.

Unless specified otherwise, continuous data were described as median (IQR (Q3: 75 percentile; Q1: 25 percentile) for skewed distributions. Categorical data were described as the number of cases (%). The statistical analysis of differences in not normally distributed variables between two independent groups was compared by the Mann–Whitney U test. The statistical analysis of differences in not normally distributed variables between three independent groups was compared by the Kruskal–Wallis test. Categorical variables were compared using Pearson’s chi-square test or Fisher’s exact test. The degree of relation between variables was evaluated with Spearman correlation analysis.

Univariate and multivariate logistic regression analyses were performed to assess the association between mortality and the risk factors findings.

First, a one-variable univariate logistic regression was used for each risk factor that was thought to be related to mortality. Each risk factor that had a *p*-value of <0.25 in the univariate variable logistic regression was included in the multivariable logistic regression model using the Enter method. The Wald statistic was used to analyze whether each independent variable was significant in the multivariable logistic regression model. The t statistic was used to analyze whether each independent variable was significant in the multivariable linear regression model.

A *p*-value of <0.05 was considered the significant level in all statistical analyses.

## 3. Results

Between 1 April 2022 and 31 December 2023, the data of 340 patients with pneumosepsis/septic shock were reviewed, and 230 patients met the inclusion criteria and were included in this study. Among these patients, 131 (57%) were male, with a median age of 73 years (range 18–95, IQR: 20) and a median BMI of 24.9 kg/m^2^ (range 13.8–52, IQR: 6). The median scores for the scoring systems calculated on the day of ICU admission were as follows: CCIS: 6, CURB-65: 3, APACHE-II: 24, and SOFA score: 7. Of the patients, 146 (63.5%) were admitted to the ICU with a diagnosis of pneumosepsis, while 84 (36.5%) were admitted with pneumonia-related septic shock. A total of 142 patients required MV, 128 patients required invasive MV, and 14 patients required non-invasive MV.

The median CRP values measured during ICU follow-up were as follows: day 1: 109.5 mg/L (range 7–478, IQR: 127), day 3: 117 mg/L (range 3–428, IQR: 120), day 7: 78 mg/L (range 3–355, IQR: 111), and day 10: 74 mg/L (range 9–359, IQR: 120). The median PCT values during ICU follow-up were as follows: day 1: 0.88 ng/mL (range 0.01–89, IQR: 3.73), day 3: 1 ng/mL (range 0.01–100, IQR: 4.75), day 7: 0.71 ng/mL (range 0.01–35, IQR: 2.36), and day 10: 1.07 ng/mL (range 0.01–67.3, IQR: 3.65). The median leukocyte counts during ICU follow-up were as follows: day 1: 11.55 × 10^3^/µL (range 1–57, IQR: 10.80), day 3: 10.80 × 10^3^/µL (range 1.7–60.4, IQR: 8.86), day 7: 10.0 × 10^3^/µL (range 2.5–31.2, IQR: 7.6), and day 10: 11.2 × 10^3^/µL (range 2.6–37.3, IQR: 9.40). Among the 230 patients, 92 (40%) had positive 1-month mortality and 118 (51.3%) had positive 3-month mortality.

In patients with 1-month mortality, the proportion of male patients, CCIS, CURB-65, APACHE II, and SOFA scores, duration of MV, incidence of septic shock, and the need for inotropic support were found to be statistically significantly higher compared with those without mortality (*p* < 0.05). Additionally, BMI and discharge rates were statistically significantly lower in patients with 1-month mortality compared with those who survived (*p* < 0.05) (Table 1).

In patients with 3-month mortality, the proportion of male patients, CCIS, CURB-65, APACHE II, SOFA scores, MV duration, incidence of septic shock, and the need for inotropic support were also found to be statistically significantly higher compared with those without mortality (*p* < 0.05). Similarly, BMI and discharge rates were statistically significantly lower in patients with 3-month mortality compared with those who survived (*p* < 0.05) (Table 1).

In patients with 1-month mortality, CRP levels on days 1 and 3, PCT levels on days 3 and 10, leukocyte counts on days 3 and 10, ALT, and total bilirubin levels were statistically significantly higher, while albumin levels were statistically significantly lower, compared with those without mortality (Table 2).

For patients with 3-month mortality, CRP levels on days 1, 3, 7, and 10, PCT levels on days 1, 3, 7, and 10, leukocyte counts on days 1, 3, 7, and 10, as well as AST, ALT, and total bilirubin levels were statistically significantly higher, while albumin levels were statistically significantly lower compared, with those without mortality (Table 2).

**Figure 2:**
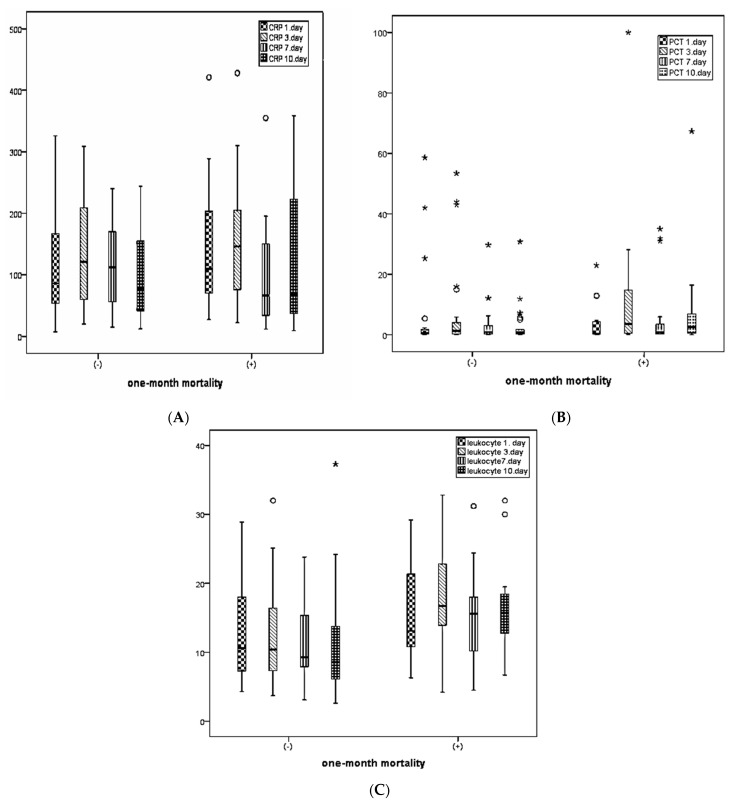
Distribution of CRP, PCT, and leukocyte values in 1-month mortality. (**A**) CRP values, (**B**) PCT values, and (**C**) leukocyte values. Outlier—○, extreme outlier—*.

Figure 2 illustrates the distribution of CRP, PCT, and leukocyte levels measured on days 1, 3, 7, and 10 between patients with and without 1-month mortality.

**Figure 3:**
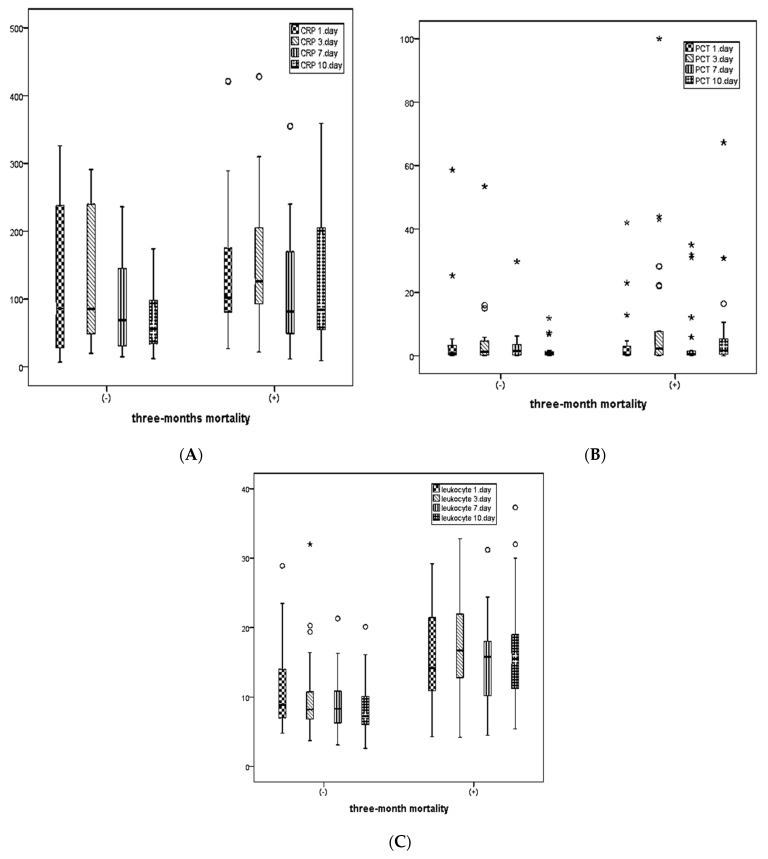
Distribution of CRP, PCT, and leukocyte values in 3-month mortality. (**A**) CRP values, (**B**) PCT values, and (**C**) leukocyte values. Outlier—○, extreme outlier—*.

Figure 3 shows the distribution of CRP, PCT, and leukocyte levels measured on days 1, 3, 7, and 10 between patients with and without 3-month mortality.

Among the 92 patients with 1-month mortality, 9 (9.8%) had an increase in only one of the CRP, PCT, or leukocyte levels on day 1, 49 (53.3%) had increases in two of these values, and 34 (37.0%) had increases in all three values. No significant relationship was found among the simultaneous increase in CRP, PCT, and leukocyte levels on day 1 and 1-month mortality between patients with and without mortality (*p* = 0.09). For the 118 patients with 3-month mortality, 9 (7.6%) had an increase in only one of the CRP, PCT, or leukocyte levels on day 1, 60 (50.8%) had increases in two of these values, and 49 (41.5%) had increases in all three values. A significant relationship was found among the simultaneous increase in CRP, PCT, and leukocyte levels on day 1 and 3-month mortality between patients with and without mortality (*p* < 0.05). The concurrent increase in any two or all three of the CRP, PCT, and leukocyte levels on day 1 was higher in patients with 3-month mortality compared with those without mortality (*p* = 0.004).

Univariate logistic regression analysis was applied to identify factors predicting 1-month mortality. Variables with a *p*-value of less than 0.05 were considered as mortality predictors. It was found that increased age, decreased BMI, male gender, higher CCIS, CURB-65, APACHE-II, and SOFA scores, shorter ICU stay, and increased leukocyte count on day 1 were associated with an increased risk of mortality (*p* < 0.05). Variables with a *p*-value of less than 0.25 in the univariate logistic regression analysis were included in the multivariate logistic regression analysis, using the Enter method. A model explaining 1-month mortality was established based on the multivariate logistic regression analysis. According to this analysis, lower BMI, male gender, higher CCIS, CURB-65, and APACHE-II scores, and shorter ICU stay were identified as factors that increased the risk of 1-month mortality (*p* < 0.05) (Table 3).

Similarly, univariate logistic regression analysis was conducted to identify factors predicting 3-month mortality. Variables with a *p*-value of less than 0.05 were considered predictors or indicators of mortality. It was found that increased age, decreased BMI, male gender, increased leukocyte count on day 1, and higher CCIS, CURB-65, APACHE-II, and SOFA scores were associated with an increased risk of 3-month mortality (*p* < 0.05). Variables with a *p*-value of less than 0.25 in the univariate logistic regression analysis were included in the multivariate logistic regression analysis, using the Enter method. A model explaining 3-month mortality was established based on the multivariate logistic regression analysis. According to this analysis, increased age, lower BMI, male gender, and higher CCIS, CURB-65, and APACHE-II scores were identified as factors that increased the risk of 3-month mortality (*p* < 0.05) (Table 4). 

## 4. Discussion

In this study examining 230 patients admitted to the ICU with a diagnosis of pneumosepsis or pneumonia-related septic shock, the primary outcome identified male gender, advanced age, low BMI, and high CCIS, CURB-65, and APACHE-II scores as significant predictors of mortality. The secondary outcome of this study highlighted that increases in CRP, PCT, and leukocyte levels, particularly on day 3, were significant for both 1-month and 3-month mortality. Additionally, among patients with 3-month mortality, the concurrent increase in any two or all three of CRP, PCT, and leukocyte levels on day 1 was higher compared with those who did not experience mortality.

A study involving 171 patients found that CRP levels measured a few days after admission, rather than initial concentrations, might be more useful in evaluating the treatment response and outcome of sepsis. CRP levels above 100 mg/L on day 3 in the ICU were found to be predictive of mortality [9]. In another study evaluating patients with severe community-acquired pneumonia in the ICU, a PCT level below 0.95 ng/mL on day 3 in intubated patients was associated with a 95% survival probability [11]. Hillas et al. found that in patients with ventilator-associated pneumonia, those who developed septic shock had higher PCT levels on both day 1 and day 4 compared with other days [12]. Similarly, our study found that PCT, CRP, and leukocyte levels on day 3 in community-acquired pneumonia were significantly higher in patients who experienced mortality within 1 and 3 months compared with those who did not. These findings suggest that these three parameters may serve as useful predictors of mortality and may be more useful for physicians to assess the treatment response and sepsis outcome in the ICU. This also highlights the importance of initiating appropriate treatment at an early stage.

Multiple studies have indicated that changes in PCT and CRP levels, rather than their absolute values, are more successful in predicting treatment response and survival [9,11,13]. A study conducted in Switzerland emphasized that the initial PCT level in community-acquired pneumonia did not improve clinical scores for predicting mortality, whereas monitoring PCT kinetics and observing decreasing levels during follow-up were more associated with improved clinical outcomes [14]. Another study found that the kinetics of PCT, CRP, and, to a lesser extent, WBC markers in community-acquired pneumonia provided additional prognostic information for mortality risk prediction [15]. Procalcitonin kinetics has been shown to be a predictor of clinical efficacy in patients with nosocomial pneumonia [16]. In our study, CRP, PCT, and leukocyte levels were found to be high on days 1, 3, 7, and 10 in patients with 3-month mortality. This indicates that persistent elevation in these values may influence long-term mortality.

In a study involving patients with severe influenza and bacterial co-infections, initial PCT and CRP levels at ICU admission were not associated with prognosis [13]. Similarly, multiple studies have confirmed that a single PCT measurement at ICU admission cannot reliably predict the prognosis of critically ill septic patients [17]. In our study, logistic regression analysis also did not find a significant relationship between CRP, PCT, and leukocyte levels on day 1 and 1-month mortality.

In patients presenting to the emergency department with suspected infection and sepsis, leukocyte, CRP, and PCT levels, when evaluated in combination with clinical severity scores, have been shown to be reliable biomarkers for prognosis [18]. The combination of PCT and CRP is particularly important in the diagnosis and prognosis of bacterial bloodstream infections [19]. In patients with severe community-acquired pneumonia, the concurrent elevation of serum PCT, leukocyte, and CRP levels has been associated with mortality [20]. It has been found that the combination of the neutrophil/lymphocyte ratio, CRP, and WBC may be helpful in early diagnosis and assessment of severity in hospital-acquired pneumonia [21]. Our study also found that 3-month mortality was higher in patients who had a concurrent increase in any two or all three of the PCT, CRP, and leukocyte levels compared with those without mortality. This supports the assumption that the assessment of multiple biomarkers may serve as a diagnostic tool in determining prognosis.

A study on patients with septic shock found that mortality was significantly higher in patients with a BMI < 25 kg/m^2^ compared with those with a BMI > 30 kg/m^2^, and lung and fungal infections were more frequently observed [22]. A meta-analysis examining the role of increased body mass index in sepsis patients also found that patients with a BMI ≥ 25 kg/m^2^ were associated with a lower risk of mortality [23]. The significantly higher incidence of sepsis in the low BMI group has been attributed to the protective effects of estrogens derived from adipocytes and the immunomodulatory effects of adiponectin [24]. In our study, the highest BMI was 52, and 37 patients had a BMI of 30 and above. No significant relationship was found between high BMI and mortality. In our study, low BMI was found to be an independent risk factor for both 1-month and 3-month mortality. Considering that obesity is a comorbidity generally associated with poor clinical outcomes, this result highlights the importance of large-scale research on the impact of increased body fat tissue on sepsis pathophysiology.

In a study involving 674 sepsis patients, advanced age, along with low BMI, was identified as an independent risk factor affecting in-hospital mortality [25]. Moreover, studies on sepsis frequently find that mortality is higher in males compared with females [2,26]. This has been attributed to the suppressive effects of male sex hormones (androgens) on cell-mediated immune responses, while female sex hormones provide a protective effect under septic conditions [27]. In our study, both advanced age and male gender were found to be independent risk factors for 1-month and 3-month mortality in patients with pneumosepsis and septic shock. Additionally, the protective effect of estrogen and other sex hormones observed in females supports the relationship between increased BMI and estrogen effects.

A study conducted in the ICUs of a tertiary hospital in China found that the APACHE-II score calculated on the first day after diagnosis was an independent risk factor for mortality [28]. Another study demonstrated that the CURB-65 score performed better than other severity scores in predicting the risk of death among septic patients [29]. In a study examining the effectiveness of CURB-65 and APACHE-II scores in assessing sepsis severity and predicting mortality, both scores were found to be successful in predicting mortality. However, since the CURB-65 score is a simple calculation, it has the advantage of being quickly calculated in crowded emergency departments, making it superior to other complex severity scores [30]. The combination of CRP, PCT, and WBC elevations with a high CURB-65 score further enhances the ability to identify patients at risk of poor clinical outcomes [15]. In our study, a significant relationship was found between increases in CURB-65 scores and elevations in CRP, PCT, and leukocyte levels at different follow-up times. Additionally, CURB-65 and APACHE-II scores were independent predictors of both 1-month and 3-month mortality in patients with pneumosepsis and septic shock. This suggests that the combined use of scoring systems and biomarkers may provide more accurate results in predicting prognosis.

In sepsis patients, the CCIS is an independent predictor of in-hospital mortality and also a predictive factor for the development of postoperative sepsis [31,32]. A study found that patients with a high CCIS were more likely to progress from bacteremia to sepsis [33]. Furthermore, it has been concluded that the CCIS is a one-year mortality predictor in patients presenting to the emergency department with severe sepsis or septic shock [34]. In our study, the CCIS was found to be a predictor of both 1-month and 3-month mortality. This supports the notion that comorbidity and advanced age worsen the general condition of patients with sepsis and septic shock.

Sepsis is an acute event and has not been associated with long-term mortality due to acute organ dysfunction. However, treatment limitation decisions related to the underlying disease also affect ICU mortality [35]. In our study, a shorter ICU stay was significantly associated with 1-month mortality. This was attributed to the acute deterioration caused by sepsis and septic shock.

Some studies have emphasized that high PCT levels in a non-septic patient may identify patients at increased risk of AKI and may even be a new biomarker for AKI [36]. A study investigating whether serum PCT levels measured at admission are a risk factor for the development of AKI in septic and non-septic patients also showed that PCT values were higher in AKI patients than in non-AKI patients, but PCT measurement at admission did not improve the prediction model for AKI [37]. In our study, although a very low positive correlation was observed between the PCT value and creatinine value, no significant relationship was found between dialysis need and PCT elevation and mortality.

Our study has several limitations. First, the retrospective and single-center design of our study limits the generalizability of the results. Second, some patients progressed to septic shock after being admitted to the ICU with a diagnosis of sepsis. Similarly, patients who were receiving monotherapy with antibiotics at the time of ICU admission and subsequently experienced worsening of their condition were switched to combination therapy. Therefore, the distinction between sepsis and septic shock and between monotherapy and combination therapy was made only on the day of admission to the ICU. The third limitation was the unclear etiology in patients who developed a dialysis indication after sepsis/septic shock. Fourth, some patients’ CRP, PCT, and leukocyte values on days 3, 7, and 10 could not be recorded because of early mortality in the ICU or transfer to an external facility.

## 5. Conclusions

In conclusion, although PCT, CRP, and leukocyte levels measured at the time of admission were not significantly associated with 1-month mortality in patients with pneumosepsis or pneumonia-related septic shock, the persistent elevation of these values, male gender, advanced age, low BMI, and high scores on the CCIS, CURB-65, and APACHE-II were significantly associated with 3-month mortality. The concurrent elevation of CRP, PCT, and leukocyte levels is a determinant in predicting long-term prognosis. Since early initiation of appropriate treatment in patients with pneumosepsis and septic shock will reduce mortality, it is extremely important to monitor the kinetics of CRP, PCT, and leukocyte values to predict prognosis and to determine the appropriate treatment rapidly. More homogeneous and large-scale prospective studies will be highly beneficial in clarifying this topic.

## Figures and Tables

**Table 1:** Evaluation of variables affecting 1-month mortality and 3-month mortality.

		1-Month Mortality			3-Month Mortality		
		(−)	(+)	*p*	(−)	(+)	*p*
		Median/IQR	Median/IQR		Median/IQR	Median/IQR	
Age (year)		72.50/19.00	74.00/18.00	0.245	71.00/18.50	74.50/19.00	0.134
BMI (kg/m^2^)		25.90/6.90	24.20/5.17	**0.001**	26.20/8.20	24.20/5.34	**<0.001**
CCIS		6.00/4.00	7.00/3.00	**<0.001**	6.00/4.00	7.00/2.00	**0.001**
CURB-65		3.00/2.00	4.00/1.00	**<0.001**	3.00/1.00	4.00/1.00	**<0.001**
APACHE II		23.00/8.00	27.00/9.00	**<0.001**	22.00/7.00	27.00/9.00	**<0.001**
SOFA		6.00/3.00	8.00/4.00	**0.001**	6.00/3.50	8.00/4.00	**<0.001**
Length of stay in ICU (day)		5.00/13.00	4.50/9.00	0.111	4.00/9.00	5.50/13.00	0.562
Duration of MV (day)		0.00/6.00	2.00/5.00	**<0.001**	0.00/4.00	2.00/7.00	**<0.001**
		**n (%)**			**n (%)**		
Gender	Male	64 (46.4%)	67 (72.8%)	**<0.001**	49 (43.8%)	82 (69.5%)	**<0.001**
	Female	74 (53.6%)	25 (27.2%)		63 (56.3%)	36 (30.5%)	
Sepsis/Septic shock	Sepsis	112(81.2%)	34 (37.0%)	**<0.001**	100 (89.3%)	46 (39.0%)	**<0.001**
	Septic shock	26 (18.8%)	58 (63.0%)		12 (10.7%)	72 (61.0%)	
Discharge	No	5 (3.6%)	75 (81.5%)	**<0.001**	2 (1.8%)	78 (66.1%)	**<0.001**
	Yes	133 (96.4%)	17 (18.5%)		110 (98.2%)	40 (33.9%)	
Need for RRT	No	113 (81.9%)	72 (78.3%)	0.497	95 (84.8%)	90 (76.3%)	0.102
	Yes	25 (18.1%)	20 (21.7%)		17 (15.2%)	28 (23.7%)	
Need for inotropic support	No	105 (76.1%)	28 (30.4%)	**<0.001**	98 (87.5%)	35 (29.7%)	**<0.001**
	Yes	33 (23.9%)	64 (69.6%)		14 (12.5%)	83 (70.3%)	
Antibiyotic	Monotherapy	68 (49.3%)	45 (48.9%)	0.957	59 (52.7%)	54 (45.8%)	0.294
	Combined therapy	70 (50.7%)	47 (51.1%)		53 (47.3%)	64 (54.2%)	

*Continuous variables are expressed as median (IQR), Mann–Whitney U test p = level of significance in bold, p < 0.05. Categorical variables are expressed as either frequency (percentage). Chi-square test p = level of significance, p < 0.05.* BMI: body mass index; CCIS: Charlson Comorbidity Index score; CURB-65: confusion, urea, respiratory rate, blood pressure, age ≥ 65; APACHE-II: Acute Physiologic and Chronic Health Evaluation-II, SOFA: Sequential Organ Failure Assessment; ICU: intensive care unit; MV: mechanical ventilation; RRT: Renal Replacement Therapy.

**Table 2:** Evaluation of laboratory values for 1-month mortality and 3-month mortality.

	1-Month Mortality			3-Month Mortality		
	(−)	(+)	*p*	(−)	(+)	*p*
	Median/IQR	Median/IQR		Median/IQR	Median/IQR	
CRP day 1 (mg/L)	98.90/138.00	128.40/125.50	**0.049**	98.00/147.50	117.00/115.00	**0.028**
CRP day 3 (mg/L)	108.00/88.50	145.00/135.00	**0.008**	99.20/94.00	134.00/129.00	**0.001**
CRP day 7 (mg/L)	78.00/88.00	68.80/161.00	0.176	68.15/104.70	92.45/131.00	**0.008**
CRP day 10 (mg/L)	78.00/113.70	68.00/186.00	0.793	56.00/64.50	84.00/150.00	**0.021**
PCT day 1 (ng/mL)	0.87/2.55	1.15/10.40	0.066	0.77/2.59	1.15/7.99	**0.019**
PCT day 3 (ng/mL)	0.50/2.59	3.40/13.30	**<0.001**	0.42/2.42	2.40/9.20	**<0.001**
PCT day 7 (ng/mL)	0.55/1.79	1.03/3.18	0.126	0.45/1.91	0.95/3.16	**0.027**
PCT day 10 (ng/mL)	0.79/1.56	2.55/6.41	**0.020**	0.79/1.19	1.75/4.87	**0.048**
Leukocyte day 1 (×10^3^ µL)	11.50/10.10	11.60/11.80	0.429	10.80/7.80	12.85/12.30	**0.049**
Leukocyte day 3 (×10^3^ µL)	10.01/6.50	13.90/11.80	**0.018**	9.55/5.30	13.80/10.90	**<0.001**
Leukocyte day 7 (×10^3^ µL)	9.40/5.00	11.70/9.36	0.062	8.90/4.00	11.85/8.60	**0.002**
Leukocyte day 10 (×10^3^ µL)	8.60/7.70	15.75/5.70	**<0.001**	7.20/4.10	15.50/7.80	**<0.001**
Hemoglobin (g/dL)	10.60/3.50	10.20/3.30	0.998	10.65/3.30	10.20/3.40	0.885
Platelet (×10^3^ µL)	216.00/120.00	221.00/174.00	0.270	217.00/102.00	208.00/167.00	0.753
GFR (mL/dk)	64.00/57.00	53.50/54.70	0.420	62.00/57.00	61.00/56.00	0.873
Creatinine (mg/dL)	1.07/1.00	1.20/1.12	0.319	1.10/1.00	1.01/1.14	0.589
BUN (mg/dL)	56.50/70.00	53.00/34.50	0.698	56.00/61.70	54.00/37.00	0.161
AST (IU/L)	22.50/18.00	23.00/40.00	0.097	19.50/14.50	25.50/25.00	**0.001**
ALT (IU/L)	14.50/14.00	18.00/45.00	**0.039**	14.00/11.00	18.00/32.00	**0.028**
Total bilirubin (mg/dL)	0.50/0.60	1.15/1.40	**<0.001**	0.50/0.50	1.00/1.10	**<0.001**
Albumin (g/L)	24.00/27.90	3.30/22.30	**<0.001**	27.00/30.25	23.45/22.40	**<0.001**

*Continuous variables are expressed as median (IQR), Mann*–*Whitney U Test p = level of significance in bold, p < 0.05*. CRP: C-reactive protein; PCT: procalcitonin; GFR: glomerular filtration rate; BUN: blood urea nitrogen; AST: aspartate aminotransferase; ALT: alanine aminotransferase.

**Table 3:** Logistic regression analysis to determine the factors affecting 1-month mortality.

	Univariate Logistic Regression					Multivariate Logistic Regression				
	Wald	*p*	OR	95% C.I. for OR		Wald	*p*	OR	95% C.I. for OR	
				Lower	Upper				Lower	Upper
Age (year)	4.180	**0.041**	1.019	1.001	1.037	3.805	0.051	0.973	0.947	1.000
BMI (kg/m^2^)	9.469	**0.002**	0.936	2.897	2.976	11.665	**0.001**	0.916	0.871	0.963
Gender (ref cat–female)	15.217	**<0.001**	3.099	1.755	5.470	14.542	**<0.001**	4.285	2.028	9.051
CCIS	12.877	**<0.001**	1.227	1.097	1.372	9.995	**0.002**	1.334	1.116	1.596
CURB-65	31.581	**<0.001**	2.600	1.863	3.629	14.525	**<0.001**	2.470	1.551	3.932
APACHE-II	20.952	**<0.001**	1.096	1.054	1.140	5.007	**0.025**	1.077	1.009	1.149
SOFA	14.854	**<0.001**	1.180	1.085	1.284	0.005	0.942	0.995	0.871	1.137
LOS ICU	5.114	**0.024**	0.962	2.931	2.995	10.329	**0.001**	0.924	0.880	0.970
Need for RRT	2.459	0.498	1.256	2.650	2.425					
Antibiyotic Mono/Comb	2.003	0.957	1.015	2.599	1.720					
CRP day 1 (mg/L)	2.427	0.119	1.002	2.999	1.005	0.005	0.943	1.000	0.996	1.004
PCT day 1 (ng/mL)	2.571	0.109	1.017	2.996	1.039	0.378	0.539	0.990	0.957	1.023
Leukocyte Day 1 (×10^3^ µL)	3.927	**0.048**	1.034	1.000	1.069	0.321	0.571	1.012	0.971	1.054

*Wald: test statistics, OR: odds ratio, CI: confidence interval. Statistically significant p-values are in bold*. BMI: body mass index; CCIS: Charlson Comorbidity Index score; CURB-65: confusion, urea, respiratory rate, blood pressure, age ≥ 65; APACHE-II: Acute Physiologic and Chronic Health Evaluation-II, SOFA: Sequential Organ Failure Assessment; LOS ICU: length of stay in intensive care unit; RRT: renal replacement therapy; Mono/Comb: monotherapy/combined therapy; CRP: C-reactive protein; PCT: procalcitonin.

**Table 4:** Logistic regression analysis to determine the factors affecting 3-month mortality.

	Univariate Logistic Regression					Multivariate Logistic Regression				
	Wald	*p*	OR	95% C.I. for OR		Wald	*p*	OR	95% C.I. for OR	
				Lower	Upper				Lower	Upper
Age (year)	4.744	**0.029**	1.019	1.002	1.036	4.626	**0.031**	0.969	0.942	0.997
BMI (kg/m^2^)	15.713	**<0.001**	0.918	0.879	0.957	17.269	**<0.001**	0.881	0.829	0.935
Gender (ref cat–female)	15.141	**<0.001**	2.929	1.705	5.032	20.324	**<0.001**	0.158	0.071	0.352
CCIS	11.803	**0.001**	1.214	1.087	1.356	5.349	**0.021**	1.261	1.036	1.535
CURB-65	48.032	**<0.001**	4.009	2.707	5.937	25.460	**<0.001**	4.081	2.363	7.047
APACHE-II	26.617	**<0.001**	1.121	1.073	1.171	8.654	**0.003**	1.105	1.034	1.182
SOFA	17.294	**<0.001**	1.217	1.110	1.336	1.473	0.225	0.913	0.787	1.058
LOS ICU	0.582	0.445	0.991	0.968	1.015					
Need for RRT	2.633	0.105	1.739	0.891	3.391	0.968	0.325	1.618	0.620	4.221
Antibiyotic Mono/Comb	1.098	0.295	1.319	0.786	2.216					
CRP day 1 (mg/L)	1.999	0.157	1.002	0.999	1.005	0.008	0.928	1.000	0.996	1.004
PCT day 1 (ng/mL)	1.184	0.277	1.012	0.991	1.033					
Leukocyte Day 1 (×10^3^ µL)	6.353	**0.012**	1.047	1.010	1.085	1.531	0.216	1.031	0.983	1.081

*Wald: test statistics, OR: odds ratio, CI: confidence interval. Statistically significant p-values are in bold.* BMI: body mass index; CCIS: Charlson Comorbidity Index score; CURB-65: confusion, urea, respiratory rate, blood pressure, age ≥ 65; APACHE-II: Acute Physiologic and Chronic Health Evaluation-II, SOFA: Sequential Organ Failure Assessment; LOS ICU: length of stay in intensive care unit; RRT: renal replacement therapy; Mono/Comb: monotherapy/combined therapy; CRP: C-reactive protein; PCT: procalcitonin.

## Data Availability

The raw data supporting the conclusions of this article will be made available by the authors on request.
